# Black soldier fly as feed ingredient for ruminants

**DOI:** 10.5713/ab.21.0460

**Published:** 2022-02-01

**Authors:** Dewi Apri Astuti, Komang Gede Wiryawan

**Affiliations:** 1 Department of Nutrition and Feed Technology, Faculty of Animal Science, IPB University, Bogor, 16680, Indonesia

**Keywords:** Black Soldier Fly, Creep Feed, Frass, Milk Replacer, Probiotic

## Abstract

This paper is a review of some experiments using black soldier fly (BSF) and its by-product to explore their nutritional value, production potential in Indonesia and its application in the ration of ruminants. Evaluation on the effect of milk replacer, creep feed containing BSF, BSF frass and the possibility to use lactic acid bacteria from BSF as probiotics are presented. Utilization of BSF larvae in milk replacer as skim and cream milk substitute showed that there were similarity on physiological, hematological status and performance of goat kids compared to those offered goat milk or commercial milk replacer. In addition, BSF larvae can be used to substitute soybean meal in the creep feed for post weaning goat kids without any differences in weight gain and blood profiles. However, utilization of BSF frass in the fattening goat ration resulted lower digestibility of dry matter and organic matter due to the chitin content in the frass. Black soldier fly larvae grown on chicken manure harbour lactic acid bacteria (LAB) which have potential as probiotics for ruminants. In general, BSF larvae has potential as ingredient for milk replacer, creep feed, fattening ration, and source of LAB for probiotics.

## INTRODUCTION

Increasing world population requires an increase of food production, including livestock production. Livestock products such as meat, milk, eggs as part of food production is the fastest growing agricultural sector. The dominant livestock types are pig with 112.33 MT, poultry 109.02 MT, and cattle, which includes beef and buffalo meat 67.99 MT representing 91.80% of meat production in the world [1]. Indonesia is the fourth most populated country in the world and also needs high livestock products especially from ruminants. Small ruminant animals such as goat and sheep are potential sources of ruminant meat, but the production and reproduction performances are still very low. To improve the animal production, it is necessary to improve feed quality offered to the animals.

Recently, there is a growing use of insects as feed ingredients due to their high nutritional content. One of well-known insect named black soldier fly (BSF) especially *Hermetia illucens*, has been more and more used commercially in feed because of easy rearing, high yield, rich nutritional value and the ability to utilize organic wastes. Black soldier fly larvae can consume substrate 25 mg up to 500 mg fresh matter per larvae per day to produce body length around 27 mm, 6 mm wide and weight 220 mg at 14 days old [2]. The nutrient composition of BSF larvae reared with mixed organic wastes ranges from 42% to 47%, 11.8% to 34.8%, 7% to 9%, and 14.6% to 15.9% for crude protein (CP), fat, crude fiber (CF), and ash, respectively [3]. Meanwhile if BSF are reared on palm oil meal, they contain 43% CP, 19.51% fat, 12.27% CF and 4.85% ash, also contain a variety of fatty acids and amino acids as presented in Tables 1 and 2 [4,5]. Based on the BSF nutrient content, they have great potential as a feed ingredient for ruminants, especially to substitute soybean meal, fish meal and antimicrobial growth promoters as they also contain antibacterial medium length chain fatty acids.

Black soldier fly larvae can grow well in harsh condition such as on food waste or animal manure, and some researchers reported that BSF larvae harbour many types of microorganisms including lactic acid bacteria (LAB) [6]. Therefore, it may be important to study the potential of those LAB as probiotics candidate to replace the use of antibiotic growth promoter especially for young ruminants. This article review describes the potential of BSF and its by-product as ingredients in milk replacer, creep feed, and growing ration of kids as well as the potential of LAB isolated from BSF larvae as probiotics for ruminants.

## THE POTENTIAL OF BSF PRODUCTION IN INDONESIA

Indonesia is an archipelago and tropical country which is very suitable for BSF production. Black soldier fly needs intensive direct ultraviolet light from the sun for mating, laying and development as a larvae. Utilization of BSF in Indonesia started in 2005. According to data of Indonesian Ministry of Fisheries in 2021, there are more than 175 BSF farmers from west (Sumatra island) to east (Papua island) with average production rate of 100 kg per day. Most of them rear BSF using organic waste and the larvae are directly fed to their fish, local poultry while very few go to ruminant feed. Only a few in the BSF industry use palm oil meal to produce BSF as an export commodity. The leftover medium after it is used for BSF larvae growth, called frass and liquor can be used for organic fertilizer. The problem with a small scale production is the price. In Indonesia, the dry BSF price is still more expensive compared to imported soybean meal, fish meal or meat bone meal. Good manufacturing practice may reduce the BSF production cost.

## APLICATION OF BLACK SOLDIER FLY LARVAE IN RUMINANT RATIONS

Black soldier fly is currently the most widely used insect in animal feed research due to its high nutritional value and cost effectiveness [7]. The demand for ruminant feed from forage and agricultural waste have been increasing every year. Originally, ruminants could survive with hay and forage, but in some cases they need additional feed or functional feed such as milk replacer for a new born, flushing diet to boost growth rate, and induce hormones during reproduction or special diet for recovering from stress. Black soldier fly with high essential amino acids, lauric acid as an antibacteria and LAB as probiotics has potential to be used as functional feed for ruminants. This statement is supported by previous study where some insects were used as potential feed ingredients for ruminants [8]. Fuctional feed is usually given for a short period to improve the condition according to the purpose of the supplementation.

The feed cost of ruminant industry usually contributes up to 40% to 60% of the production cost, and the proportion of protein accounts for over 15% of total feed cost [9]. Many kinds of protein feed sources such as legumes, grains and animal waste can be used as part of the ration. For an eco-friendly industry, there is a growing interest for using insects as a protein source in livestock, therefore there is an increasing use of BSF as a source of protein [10]. Some part of BSF products can be used for functional feed, such as dry BSF, oil, meal, frass, chitin and LAB from BSF larvae digestive tracts. The application of BSF and its by-products in ruminants has carried out over the last five years at the IPB University, Indonesia.

### Utilization of black soldier fly for milk replacer

Milk replacer is formulated from high quality ingredients to provide the nutrient requirements for young animals. Milk replacer is usually given to the pre-weaning animals to replace the mother’s milk which can be sold by the farmer. The quality of milk replacer should be more or less similar to the original milk, and the quality of milk replacer should contain 20% to 24% protein, 20% to 30% fat, 1% calcium and 0.8% phosphorous [11]. Most milk replacers are dry powders that have to be reconstituted to a liquid by mixing with warm water. Some formulas of milk replacer have been made containing source of protein from cricket meal for goat kids [12], gluten hydrolyzed for calves, and puffed corn or puffed full fat soybean for lambs. An experiment was designed to compare the effectiveness of goat milk, commercial milk replacer and BSF milk replacer on pre-weaning goat kids. The milk replacer was formulated containing 30% of fourteen days old BSF larvae meal mixed with egg flour, casein, full cream, wheat flour and some other minerals and vitamins. The milk replacer was offered to the goat kids 6 to 8 times a day until 5 weeks old and then continued until 8 week together with creep feed supplementation to imporve the rumen function development. The result showed that the dry matter and protein consumption of BSF milk replacer were the same compared to consumed goat milk, however fat consumption of BSF milk replacer was significantly higher (p<0.05) than kids consumed from goat milk (Table 3). Dry matter intake of goat kids fed milk replacer containing cricket meal is around 3% to 4% of body weight [12]. The palatability of new milk replacer with specific ingredients is important to be evaluated. Texture, smell and taste are some indicators which affect the palatability. According to Nutrient Research Council [13] goats are animals which are very sensitive to selecting feed (browsers). There is a decreasing BSF milk replacer intake compared to the commercial milk replacer and goat milk during the initial four weeks. The low initial intake of BSF milk replacer is due to the adaptation period and high fat content. Feeding with BSF milk replacer increased fat intake five times compared to goat milk. In this experiment, creep feeding containing BSF larvae supplementation was started from fifth week of rearing, and it showed good palatability to pre-weaning kids. Milk replacer containing cricket meal improved the growth rate of kids similar to those offered with goat milk treatment [12]. The body weight gain of goat kids fed milk replacer containing BSF meal is the same as goat kids body weight gain fed with milk replacer containing cricket meal, which is around 157 g/d. The final body weight of weaning goats fed with goat milk is around 14 kg, however weaning kids fed with milk replacer containing BSF have body weight around 12.5 kg. The weaning weight of local Etawah crossbred with natural mating is around 11.1 kg [14]. Meanwhile Lu [15] stated that weaning weight depends on some factors such genetic, weaning age, health status, quality and quantity of mother’s milk, litter size and feeding management. Data of the physiological status such as heart rate, respiration, and body temperature were the same in all treatments and in the range of normal condition. Milk replacer containing BSF larvae meal apparently has no negative effect on the physiological status of the goat kids. Studies using BSF larvae in the milk replacer show that feed conversion is 3.67 and higher than kids consumed goat milk (1.69). Energy and protein status are closely related to body weight gain and feed conversion ratio [16].

### Utilization of black soldier fly larvae for creep feed

Creep feeding is a method of supplementing the diet of young livestock by offering feed to animals who are still nursing. Creep feeding is usually offered when lambs or kids are still nursing to provide additional nutrients to support animal growth [17]. Many studies used creep feed supplementation especially in calves to stimulate the rumen development and improve the growth rate. A trial was designed on post-weaning two-month-old Ettawah crossbred goats fed with creep feed for three months. The control diet formulated using 30% soybean meal without BSF larvae, meanwhile the other two creep feed treatments were formulated containing 15% and 30% of BSF larvae, respectively. Data showed that the physiological and hematological status of growing goats were similar in all feeding treatments and it was in the normal range. The performance of those five-month-old growing kids was good with final body weight around 20 kg. The growing goat kids fed cricket creep feed had higher average daily gain (123 g/d) [12] compared to the BSF creep feed treatment (89 g/d). It is reported that kids with intensive management could increase growth rate with consumption of 230 g/d of creep feed containing 14% CP based alfalfa [18]. Other experiments reported that with the supplementation of 18% CP creep feed, the consumption is around 152 g/d and no difference in weight gain [19]. Based on Beesigamukama et al [20] requirement for growing kids with 150 g/d weight gain needed 4.81% dry matter intake of body weight, 86 g/d of protein intake and 0.43 kg/d of total digestible nutrient (TDN). Important variable that affects the performance of kids is not only the quality of ration but also the amount of feed intake. Insects with high protein and fat content show potential to be used in a creep feeding program. The best insects with high essential amino acids content, such as BSF, can potentially be used in creep feed.

### Utilization of frass black soldier fly in the growing goat ration

Frass is the by-product of the larval meal industry that includes larval waste, exoskeleton sheds and residual feed ingredients. The quality of frass depends on the quality of medium and the amount of chitin content. In some reasearch which used organic waste medium, the frass contained around 15% CP and 25% CF with low TDN value. Information regarding BSF frass utilization for ruminant ration is still very rare. Frass mainly goes to the organic farm as a good quality fertilizer [21] or it is used for protein source in catfish [22]. Based on the nutrient quality of frass, it is possible to use it as an ingredient for ruminants. Black soldier fly frass from palm oil meal medium contains 19% CP, 21% CF and 68% TDN, so it can be used as protein source or even for fibre source for ruminants.

An experiment was done in our laboratory using frass derived from BSF leftover medium was compared with the use of commercial concentrate on growing male Ettawah goats. The ration was formulated containing 30% BSF frass from palm oil meal medium as a concentrate diet with 14% CP and 68% TDN, whereas the commercial concentrate was formulated in the same quality. Data showed that there were no significant differences of nutrient intake in both treatments, meanwhile the digestibility of dry matter, organic matter, nitrogen free extract, and TDN were significantly lower (p<0.05) compared to the commercial diet (Table 4). This condition may be due to the high content of undigested fibre and chitin in frass. Data from the *in vivo* experiment showed similar results as those of the *in vitro* experiment.

### Potential of lactic acid bacteria from black soldier fly larvae as probiotics in ruminants

Probiotics are defined as live microorganisms, providing health benefits for the host, when they are administered in adequate amounts FAO/WHO [23]. Lactic acid bacteria are commonly used as probiotics to replace antibiotic growth promoters against pathogenic bacteria due to their production of organic acids, hydrogen peroxide, and bacteriocins [24–26]. The application of LAB probiotics in young ruminants may reduce the incidence of diarrhea as well as improve body weight gain and feed efficiency. Holstein calves supplemented with *L. acidophilus* 27SC had significantly higher colony counts of lactobacilli in feces compared to calves fed a control diet. As a result, calves fed *L. acidophilus* 27SC showed significant differences in scour index during weeks 5, 7, and 8 compared with calves fed a control diet and, during weeks 7 and 8 compared with calves fed a mixed lactobacilli diet [27]. The effects of oral administration lactic acid producing bacteria of *Bifidobacterium pseudolongum* or *L. acidophilus* on newborn calves were also investigated [28]. Oral administration of the two types of LAB improved body weight gain and feed efficiency, and reduced frequencies of diarrheacompared to calves that did not receive LAB. In another investigation using the addition of *L. mucosae* and cell-free supernatant during the *in vitro* fermentation of dried brewers grain increased the volatile fatty acids production, but had no effect on dry matter and organic matter digestibility. Furthermore, the addition of *L. mucosae* can also increase the total bacterial population but have no significant effect on the total microbial diversity [29].

Lactic acid bacteria can be isolated from a different variety of sources such as soil, flowers, fruits, fermented foods, and animal digestive tracks. Recently, there is a fast growing interest on the use of insects especially BSF (*Hermetia illucens*) larvae as animal feed due to its high protein and lipids content. Black soldier fly larvae convert different kinds of organic wastes such as food waste, and animal manure [30,31]. Black soldier fly larvae can survive under harsh conditions, so they may possess some good bacteria in their intestines that can compete with pathogenic bacteria. Therefore, research has been conducted in our laboratory to isolate and characterize LAB that can be used as a probiotics candidate in ruminant animals.

**Figure 1:**
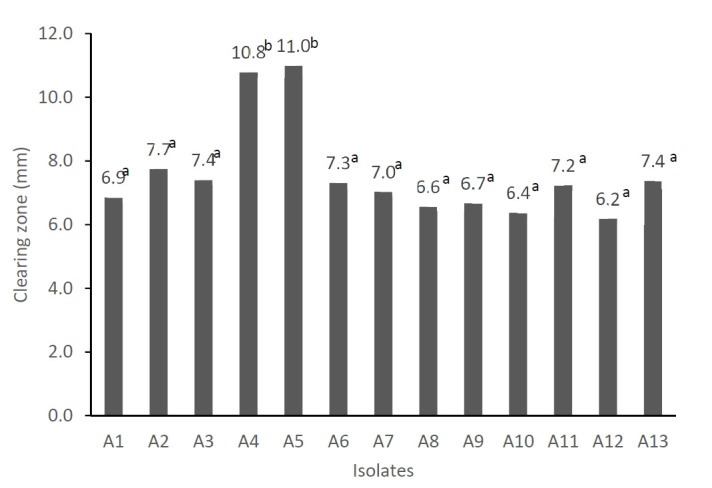
Clearing zone growth inhibition of *Escherichia coli* by lactic acid bacteria isolated from black soldier fly larvae. ^a,b^ Different superscript means significant different (p<0.05).

The results of our experiment showed that there were 13 LAB isolates which have different colors and morphologies of colonies and have characteristics of gram positive, catalase negative, and cocci cell morphology. All isolates produced organic acids as shown by low pH (ranged from 4.4 to 4.6) after 24 hours incubation in the De Man, Rogosa, and Sharpe (MRS) medium (Table 5). In addition, all isolates were able to inhibit the growth of *Escherichia coli* (*E. coli*) as shown by the clearing zone produced using agar well diffusion method [32]. Figure 1 shows the diameter of the clearing zone of *E. coli* treated with cell-free supernatant of isolates. The diameter of clearing zone ranged from 6.18 mm to 10.98 mm. The isolates A4 and A5 had significantly higher (p<0.05) inhibitory activity on *E. coli* growth compared to the other isolates. The inhibitory activity of the LAB isolates on *E. coli* in this experiment was lower compared to those LAB isolated from asam durian [33]. Production of organic acids and the ability of LAB in inhibiting the growth of pathogenic bacteria such as *E. coli* is an important factor in selecting probiotic candidates because the probiotics should be able to compete and eliminate the pathogenic bacteria in the intestines. However, it is not clear yet whether the inhibition of *E. coli* growth is only caused by organic acids or there may be other antipathogenic compounds such as bacteriocin produced. Further study is required to evaluate the production of bacteriocin.

Three isoltaes (A5, A11, and A13) which have different colony morphology and diameter of clearing zone were further tested for their ability to survive in intestinal conditions such as different pH [34] and bile salt 0.5% [35]. The results show that the three isolates were able to survive at three different pH (2, 4, and 6) (Table 6). The survival rate of LAB isolates at pH 2, 4, and 6 ranged from 73.41% to 77.13%, 80.55% to 86.28%, and, 81.23% to 94.69%, respectively. The survival rate of the isolate A5 was consistently higher (p<0.05) than that of isolate A13 in all pH conditions and was higher (p<0.05) than that of isolate A11 at pH 4 and pH 6. Isolate A5 also had higher survival rate at pH 2 compared to the survival rate of LAB isolated from soil reported by [36], but it was lower than the survival rate of LAB isolated from asam durian [33]. The LAB can survive in the acid condition due to the ability of LAB to protect membrane damages from low extracellular pH [37]. Meanwhile, the survival rate of the three LAB isolates on 0.5% bile salts ranged from 75.35% to 78.53% and there were no significant different amongst the three isolates (Table 6).

One important criteria for selecting LAB as probiotic candidates is the ability of the bacteria to adhere on the mucosal surface of the intestines. It means that the bacteria should be able to recognize the receptor availbale on the surface of intestines and form biofilm to colonize the intestines and compete with pathogenic bacteria. The method used to evaluate the adherence of LAB on the intestines is the stainless steel method [37]. The results showed that the three LAB isolates were able to adhere to the surface of stainless steel. The percentage of adhesion was ranged from 57.08% to 60.04% and there was no significant different amongst the three isolates (Table 7). The percentage of isolates adhering in this experiment was lower compared to that reported by Dewanti and Wong [38], who found that the percentage of adhesion was between 81% to 94%, but it was higher to that reported by Babot et al [39]. The differences in adhesion abilities of strains are specific and depend on physiology of cell and composition of cell wall [40].

## CONCLUSION

Black soldier fly larvae harvested on day 15th has potential to be used as an ingredient in the ruminant rations. The milk replacer and creep feed containing BSF larvae, and also concentrate containing BSF frass could improve performance of pre- and post-weaning and growing goats. BSF larvae grown on chicken manure harbor LAB which are potentially probiotic candidates.

## Figures and Tables

**Table 1:** Fatty acid composition of 15-day-old black soldier fly larvae reared on organic waste

Parameters	Black soldier fly	

	Raw	Steam
Fat content (%)	38.09	27.49
Saturated fatty acid (%)		
Capric acid (C10:0)	0.84	0.81
Lauric acid (C12:0)	40.29	49.18
Tridecanoic acid (C13:0)	0.02	0.03
Myristic acid (C14:0)	6.76	8.09
Pentadecanoic acid (C15:0)	0.12	2.70
Palmitic acid (C16:0)	9.99	8.53
Heptadecanoic acid (C17:0)	0.11	0.19
Stearic acid (C18:0)	1.27	1.42
Arachidic acid (C20:0)	0.04	0.05
Behenic acid (C22:0)	0.02	0.04
Unsaturated fatty acid (%)		
Myristoleic acid (C14:1)	0.16	0.23
Palmitoleic acid (C16:1)	2.07	2.70
Cis-10-Heptadecanoic acid (C17:1)	0.00	0.24
Elaidic acid (C18:2n9t)	0.30	0.29
Oleic acid (C18:1n9c)	7.99	5.94
Linolelaidic acid (C18:2n9t)	0.00	1.41
Linoleic acid (C18:2n6c)	4.02	0.03
v-Linolenic acid (C18:3n6)	0.00	0.00
Cis-11,14-Eicosedienoic acid (C20:2)	0.02	0.03
Cis-8,11,14-Eicosetrienoic acid (C20:3n6)	0.03	0.02
Total fatty acid (%)	74.04	79.41

Source: Harlystiarini et al [5].

**Table 2:** Analyzed amino acids content of black soldier fly larvae reared on palm oil meal

Amino acid	Percent of total amino acid
Histidine	0.83
Threonine	1.40
Arginine	2.26
Tyrosine	2.83
Methionine	0.78
Valine	2.76
Phenylalanine	2.02
Isoleucine	2.17
Leucine	2.95
Lysine	2.37
Serine	1.45
Glysine	1.42
Alanine	2.47

Source: Harlystiarini et al [5].

**Table 3:** Dry matter and protein consumption of kids fed milk replacer containing black soldier fly larvae meal

Consumption	Goat milk	Commercial MR	BSF MR	SEM
Week 1 to 4 (MR)				
Dry matter (g/d)	151.0^a^	208.0^b^	169.8^a^	14.0
Protein (g/d)	30.2	33.9	31.8	2.0
Fat (g/d)	6.3^a^	33.9^b^	33.8^b^	2.0
Week 5 to 8 (MR)				
Dry matter (g/d)	160.4^a^	210.4^b^	172.0^a^	13.0
Protein (g/d)	31.6	33.7	32.2	2.1
Fat (g/d)	6.7^a^	33.7^b^	34.2^b^	2.5
Week 5 to 8 (CF)				
Dry matter (g/d)	93.7	121.1	84.9	41.0
Protein (g/d)	15.2	19.7	13.8	6.7
Fat (g/d)	1.8	2.4	1.7	0.4

MR, milk replacer; BSF, black soldier fly; SEM, standard error of mean; CF, creep feed.

a,b Different superscript with lowercase in the same raw is significant different at (p<0.05).

Source: Astuti and Komalasari [4].

**Table 4:** Nutrient digestibility of growing goat fed black soldier fly frass

Nutrient digestibility	Concentrate commercial	Concentrate BSF frass	SEM
Dry matter (%)	74.1^b^	66.5^a^	1.9
Organic matter (%)	75.7^b^	67.4^a^	1.8
Crude protein (%)	69.5	66.5	2.6
Fat (%)	92.1	91.2	2.3
Crude fibre (%)	56.0	50.0	6.5
NFE (%)	82.0^b^	73.7^a^	2.1
TDN (%)	80.0^b^	74.4^a^	1.0

BSF, black soldier fly; SEM, standard error of mean; NFE, nitrogen free extract; TDN, total digestible nutrients.

a,b Different superscript with lowercase in the same raw is significant different at (p<0.05).

**Table 5:** pH of lactic acid bacteria (isolated from black soldier fly) after 24 hours incubation in De Man, Rogosa and Sharpe medium

Isolate	A1	A2	A3	A4	A5	A6	A7	A8	A9	A10	A11	A12	A13
pH	4.5	4.6	4.5	4.6	4.6	4.6	4.5	4.6	4.6	4.6	4.6	4.4	4.6

**Table 6:** Percentage of lactic acid bacteria (isolated from black soldier fly larvae) survival at different pH and 0.5% bile salts

Isolates	Survival (%)			

	pH 2	pH 4	pH 6	Bile salts 0.5%
A13	73.4^a^	82.0^a^	81.2^a^	76.8
A11	77.1^b^	80.6^a^	93.0^b^	78.5
A5	75.8^b^	86.3^b^	94.7^b^	75.4
SEM	1.1	1.0	4.2	1.3
p-value	0.01	0.05	<0.01	0.27

SEM, standard error of mean.

a,b Different superscript in the same column is significant difference at (p<0.05).

**Table 7:** Number and percentage of lactic acid bacteria (isolated from black soldier fly larvae) adhesion on the surface of stainless steel

Isolates	Initial population (log cell/cm^2^)	Cell adhesion (log cell/cm^2^)	Percentage of adhesion
A13	8.96	5.38	60.04
A11	8.90	5.08	57.08
A5	8.81	5.15	58.46
